# Assessment of a Small Molecule Synthetic Lignan in Enhancing Oxidative Balance and Decreasing Lipid Accumulation in Human Retinal Pigment Epithelia

**DOI:** 10.3390/ijms22115764

**Published:** 2021-05-28

**Authors:** Anuradha Dhingra, Rachel C. Sharp, Taewan Kim, Anatoliy V. Popov, Gui-Shuang Ying, Ralph A. Pietrofesa, Kyewon Park, Melpo Christofidou-Solomidou, Kathleen Boesze-Battaglia

**Affiliations:** 1 Department of Basic and Translational Sciences, School of Dental Medicine, University of Pennsylvania, Philadelphia, PA 19104, USA; dhingra@upenn.edu (A.D.); rachelcsharp3@gmail.com (R.C.S.); 2 Department of Periodontics, School of Dental Medicine, University of Pennsylvania, Philadelphia, PA 19104, USA; taewank@dental.upenn.edu; 3 Department of Radiology, Perelman School of Medicine, University of Pennsylvania, Philadelphia, PA 19104, USA; avpopov@pennmedicine.upenn.edu; 4 Center for Preventive Ophthalmology and Biostatistics, Perelman School of Medicine, University of Pennsylvania, Philadelphia, PA 19104, USA; gsying@pennmedicine.upenn.edu (G.-S.Y.); kyewpark@pennmedicine.upenn.edu (K.P.); 5 Department of Medicine, School of Medicine, University of Pennsylvania, Philadelphia, PA 19104, USA; ralphpietrofesa@gmail.com (R.A.P.); melpo@pennmedicine.upenn.edu (M.C.-S.)

**Keywords:** antioxidant, inflammation, retina, ketogenesis, LGM2605, oxidative stress, lipid steatosis, small molecule therapeutics

## Abstract

Visual function depends on the intimate structural, functional and metabolic interactions between the retinal pigment epithelium (RPE) and the neural retina. The daily phagocytosis of the photoreceptor outer segment tips by the overlaying RPE provides essential nutrients for the RPE itself and photoreceptors through intricate metabolic synergy. Age-related retinal changes are often characterized by metabolic dysregulation contributing to increased lipid accumulation and peroxidation as well as the release of proinflammatory cytokines. LGM2605 is a synthetic lignan secoisolariciresinol diglucoside (SDG) with free radical scavenging, antioxidant and anti-inflammatory properties demonstrated in diverse in vitro and in vivo inflammatory disease models. In these studies, we tested the hypothesis that LGM2605 may be an attractive small-scale therapeutic that protects RPE against inflammation and restores its metabolic capacity under lipid overload. Using an in vitro model in which loss of the autophagy protein, LC3B, results in defective phagosome degradation and metabolic dysregulation, we show that lipid overload results in increased gasdermin cleavage, IL-1 β release, lipid accumulation and decreased oxidative capacity. The addition of LGM2605 resulted in enhanced mitochondrial capacity, decreased lipid accumulation and amelioration of IL-1 β release in a model of defective lipid homeostasis. Collectively, these studies suggest that lipid overload decreases mitochondrial function and increases the inflammatory response, with LGM2605 acting as a protective agent.

## 1. Introduction

Visual function depends on the intimate structural, functional and metabolic interactions between the retinal pigment epithelium (RPE) and the neural retina (NR). In the aging human eye, oxidative damage, metabolic dysregulation and accumulation of pro-oxidant compounds cause the functional decline of the RPE, which contributes to age-related macular degeneration (AMD) [1,2,3]. AMD is a late-onset, progressive disease that generally affects individuals over the age of 60 [4]. It is the major cause of vision loss in the elderly, affecting over 2 million people in the US. With the aging of “baby-boomers”, the number of AMD cases is predicted to double by the year 2050 [4]. Uncompensated oxidative stress is a major contributor to RPE dysfunction and cell injury, and in AMD is a known risk factor in disease progression. AMD donor eyes have higher levels of oxidatively modified proteins, DNA and lipids [5]. In animal models of chronic oxidative stress, the retina and RPE develop pathologic lesions characteristic of early AMD [6,7]. RPE are at high risk for oxidative stress as they constantly manage reactive oxygen species resulting from an oxygen-rich environment, high metabolic activity and high flux of polyunsaturated fatty acids as well as exposure to the oxidizing effects of blue light [8,9]. Undegraded lipids serve as substrates for peroxidation reactions in this oxidative environment [9,10].

Over time, RPE, the single layer of epithelia, suffers from cumulative oxidative stress due to a combination of normal physiological as well as environmental factors. In this context, it is essential to understand the multifunctional role played by the RPE in retinal homeostasis. RPE function includes light absorption, heat exchange, Vitamin A metabolism, outer blood retinal barrier nutrient exchange and maintenance of chorio-capillaries [11]. Perhaps one of the most critical functions in maintaining photoreceptor physiological function is the daily renewal of photoreceptor constituents. Photoreceptor rods and cones as well as the overlying RPE are terminally differentiated, thus, to maintain structure and function, the photoreceptors rely on the diurnal phagocytosis and digestion of photoreceptor outer segment tips (OS) by the RPE. In the human retina, we estimate that each RPE ingests ~0.08–0.15 pmoles of fatty acid for fatty acid oxidation [12]. The daily phagocytosis and subsequent degradation of the lipid-rich OSs provide energy to the RPE through mitochondrial fatty acid oxidation and generate ketones bodies for use by the photoreceptors [13,14]. Because of this critical and continuous dependence of photoreceptors on RPE, any functional deficiency in RPE ultimately harms photoreceptors and impairs vision. Defects in phagocytic function and fatty acid oxidative capacity phenocopies aspects of aging and age-related retinal disease. These conditions are characterized by degeneration of the RPE, which includes the formation of atrophic patches of RPE and subretinal migration of activated microglial cells as well as sub-RPE deposition of inflammatory and oxidatively damaged proteins [3,11,15,16,17,18].

Virtually all cell types in the eye rely on one or more aspects of autophagy to maintain structure and/or normal physiological function [11,19]. Autophagy is also a critical regulator of RPE homeostasis, playing an important role in protection against oxidative stress [20,21,22]. Mutations in or loss of specific autophagy components are associated with the accumulation of proteins and damaged organelles, a common characteristic of the aging RPE as well as of AMD [23,24,25,26]. The RPE utilizes autophagy proteins, specifically LC3B, in the degradation of ingested photoreceptor outer segments, similar to the phagocytic function in macrophages and dendritic cells [27,28,29,30,31]. The daily burst of OS phagocytosis is accompanied by an increase in LC3, levels of which fluctuate over the 12 h light/dark cycle [32,33]. The RPE relies on LC3 to maintain functional mitochondria and peroxisomes [20,34] and as a critical component of a phagocytosis degradative pathway termed LC3-associated phagocytosis (LAP) [16,29,30,31,32]. The RPE has adapted LAP to eliminate stressors, including the daily ingestion of lipids and proteins in the form of OS tips [14,16,32]. LC3B-associated processes play a critical role in visual pigment regeneration and phagosome degradation [16,28,32], with loss of LAP resulting in decreased retinal function [16] and dysregulated lipid metabolism [14]. Our own previous studies established that loss of the autophagy protein LC3B leads to lipid accumulation, peroxidation, ketogenic insufficiency and induction of a proinflammatory microenvironment [27]. Collectively, such dysregulation contributed to the loss of both retinal and RPE function as assessed by ERG [27].

A unique synthetic lignan secoisolariciresinol diglucoside (SDG), LGM2605, a small molecule therapeutic, also restores mitochondrial homeostasis [35]. SDG has potent free radical scavenging and antioxidant properties and confirms its DNA radioprotective properties in cell-free systems [36] as well as in cells [37,38]. In preclinical models in mice [35,39,40,41], non-human primates [42] and in an ex vivo lung organ culture model of proton-irradiated human lung, LGM2605 [43] has been shown to be highly efficacious. LGM2605 exhibits free radical scavenging, antioxidant and anti-inflammatory properties in diverse inflammatory cells (Rom, 2018 #43; Kokkinaki, 2019 #38; Christofidou-Solomidou, 2012 #55; Mishra, 2013 #48; Velalopoulou, 2017 #47; Velalopoulou, 2015 #42; Pietrofesa, 2016 #62; Pietrofesa, 2017 #54). Most recently, LGM2605 has been shown to improve metabolic function in cardiomyocytes [35].

In these studies, we tested the hypothesis that LGM2605 may be an attractive small-scale therapeutic that restores metabolic capacity to RPE under lipid overload and protects against inflammation. Using an in vitro model of defective phagosome degradation and metabolic dysregulation, we show that lipid overload results in increased gasdermin cleavage, IL-1 β release, lipid accumulation and decreased oxidative capacity. The addition of LGM2605 resulted in enhanced mitochondrial capacity, decreased lipid accumulation and amelioration of IL-1 β release. Collectively, these studies suggest that lipid overload increases the inflammatory response and mitochondrial overload, with LGM2605 acting as a protective agent.

## 2. Materials and Methods

### 2.1. Materials

The following antibodies were used for immunoblotting (dilution; company information): mouse anti-β-actin (1:5000; A2228; Sigma-Aldrich, St. Louis, MI, USA), mouse anti-opsin mAb 4D2 (1:1000; a generous gift from Dr. R. Molday, University of British Columbia, Vancouver, BC, Canada), rabbit anti-gasdermin (GSDRM) (1:1000, NBP2-33422; Novus, Littleton, CO, USA), goat anti-mouse and goat anti-rabbit HRP-conjugated secondary antibody (1:3000; 32430 and 31462; Invitrogen, Carlsbad, CA, USA). Bovine photoreceptor outer segments (OS) were from Invision Bioresources (Seattle, WA, USA).

We independently generated synthetic SDG (referred to as LGM2605 in the literature), as previously described [44]. Briefly, secoisolariciresinol diglucosides (*S*,*S*)-SDG (the major isomer in whole grain flaxseed) and (*R*,*R*)-SDG (the minor isomer in whole grain flaxseed) were synthesized from vanillin via secoisolariciresinol and glucosyl donor (perbenzoyl-protected trichloacetimidate under the influence of TMSOTf) through a concise route involving chromatographic separation of diastereomeric diglucoside derivatives (Chemveda Life Sciences Inc., Hyderabad, India). Absorbance spectra of 36mM LGM2605 measured using a SpectraMax M5 fluorescent plate reader (Molecular Devices, San Jose, CA, USA).

### 2.2. Generation of RPE^-LC3B^

Prepackaged lentiviral particles that either encoded a nontargeting shRNA (negative shRNA; sc-108080) or sequences specifically targeting the human gene MAP1LC3B (sc-43390-V) were purchased from a commercial provider (Santa Cruz Biotechnology, Dallas, TX, USA). LC3B knockdown cells were generated by transducing ARPE-19 with *MAP1LC3B* shRNA lentiviral particles, sc-43390-V. ARPE-19 cells were passaged and subsequently (within 24 h) transduced with the shRNA lentiviral particles with a multiplicity of infection (MOI) of five in the presence of 8 μg/mL hexadimethrine bromide and incubated at 37 °C for 18–20 h [45]. Fresh media was added and cells were incubated overnight. Media were then replaced with media containing 2 μg/mL puromycin for selection purposes and changed every 3–4 days until resistant colonies could be identified. The control cell lines were generated by transducing ARPE-19 cells with negative shRNA, sc-108080. Knockdown of the *MAP1LC3B* gene was confirmed by real-time polymerase chain reaction and of LC3B protein by Western blot analysis.

### 2.3. Maintenance of RPE Cell Culture

ARPE-19 cells—(CRL-2302, ATCC) were maintained as we have described [46]. Polarized ARPE (or RPE), RPE^-MREG^ and RPE^-LC3B^ were cultured as described [14,45]. In brief, cells were grown on Transwell filters (12-well, 0.4-µm pore size) maintained in DMEM/F12 with 1% fetal bovine serum (Sigma, St. Louis, MI, USA) and 5% penicillin-streptomycin (Life Technologies Inc., Carlsbad, CA, USA) or on glass-bottom dishes at 37 °C, 5% CO_2_. Individual filters were seeded with 1.6 × 10^5^ cells/well in a total volume of 0.5 mL in the apical chamber and 1.5 mL of medium in the basal chamber, with the medium changed twice weekly beginning the day after plating.

### 2.4. Cell Stress Studies

RPE and RPE^-LC3B^ cells were treated with H_2_O_2_ (200 µM or 600 µM) and 10 ng/mL TNF-α to induce oxidative stress, as described previously [47,48]. Briefly, cells were plated on 12 well plates 48 h prior to the start of the experiment. On day 2, cells were treated with H_2_O_2_ and 10ng/mL TNF-α for 6 min. Stressed cells were washed and subsequently fed OS, at a ratio of 10:1 (10 particles/cell). Supernatants and cell lysates were collected 10 h and 24 h after treatment. In some studies, the cells were pretreated with LGM2605 (50–200 µM) in serum-free media for 30 min prior to H_2_O_2_ + TNF-α stress induction.

### 2.5. OS Phagocytic Challenge and LGM2605 Treatment

#### 2.5.1. Challenge with Photoreceptor Outer Segments (OS)

RPE and RPE^-LC3B^ cells were challenged with OS using either a single pulse/chase challenge or multiple pulse/chase challenges over a 5–7 day period. Pulse/chase studies were begun with the removal of the existing media and cells were rinsed three times with growth media. OS at a ratio of 10:1 (10 particles/cell) were added to the apical chamber in fresh growth media and incubated at 37 °C for 2 h (pulse). After 2 h pulse, cells were rinsed three times with growth media, fresh growth media was added, and phagosome degradation was allowed to continue for up to 24 h (chase) [14,32]. The assay was terminated at designated time points with the removal of apical supernatant media, cells washed three times (37 °C PBS-CM), and filters immediately processed for immunofluorescence microscopy or lysates prepared for western blot analysis or gene expression studies. These samples were collected at the times indicated in the Figure Legends.

In our long-term OS challenge studies, a pulse/chase series was repeated daily for 5–7 days, essentially as described in [49]. In those studies, on experimental day 0, the culture medium was removed from 3–5 wells and replaced either with medium or with OS at a ratio of 10:1 (10 particles/cell) in fresh growth media and incubated at 37 °C for 2 h (pulse). Cells were washed thoroughly with an isotonic solution to remove non internalized OSs, followed by a 2 h chase in media. In experiments using LGM2605, cells were incubated with LGM2605 (100 µM in serum-free media) for 30 min daily and rinsed with PBS prior to the addition of OSs. At the end of the long-term OS challenge, as indicated in the individual Figure legends, cells were analyzed for neutral lipid accumulation (BODIPY™493/503), lipid peroxidation adducts (4-HNE), IL-1 β secretion, cell integrity, (transepithelial resistance, TER) and actin staining, as well as mitochondrial function (βHB). All assays are described in detail below.

#### 2.5.2. Treatment with LGM2605

Cells were pretreated with increasing concentrations of LGM2605 (50–200 μM) in serum-free media for 30 min prior to either H_2_O_2_ + TNF-α stress induction or the addition of OS. In brief, lyophilized LMG2605 was freshly resuspended before each experiment, according to packaging instructions.

### 2.6. RNA Isolation and Gene Expression Analysis

Total RNA was isolated using a RNeasy Plus Mini Kit and quantitative polymerase chain reaction (qPCR) analysis was performed, as previously described [38,43,50,51]. Total RNA was quantified using a NanoDrop 2000 apparatus (Thermo Fisher Scientific, Waltham, MA, USA). Reverse transcription of RNA to cDNA was then performed on a Veriti^®^ Thermal Cycler using the High Capacity RNA-to-cDNA Kit (Applied Biosystems, Thermo Fisher Scientific, Waltham, MA, USA). qPCR was performed using individual TaqMan^®^ Probe-Based Gene Expression Assays (Applied Biosystems, Thermo Fisher Scientific, Waltham, MA, USA). Individual TaqMan gene expression assays were selected for nuclear factor (erythroid-derived 2)-like 2 (Nrf2) regulated genes, NADPH: quinone oxidoreductase-1 (NQO1), glutathione S-transferase mu 1 (GSTM1), and heme oxygenase-1 (HO-1). qPCR was performed using 50 ng of cDNA per reaction well on an Applied Biosystems QuantStudio 6 Flex Real-Time PCR System (Applied Biosystems, Thermo Fisher Scientific, Waltham, MA, USA). Gene expression data are presented normalized to the housekeeping gene glyceraldehyde 3-phosphate dehydrogenase (GAPDH). Gene expression analysis was also performed using two additional housekeeping genes, 18S ribosomal RNA (18S rRNA) and β-actin for validation. All data were calibrated to the control, untreated samples (RPE Ctrl) according to the ΔΔCT method as previously described [52].

### 2.7. Immunoblotting

Cell lysates were prepared in RIPA buffer with 1% protease inhibitor mixture (Sigma; P8340, St. Louis, MI, USA) and 2% phosphatase inhibitor mixture 2 (Sigma; P5726, St. Louis, MI, USA). 10–15 μg of the protein per sample was separated on 4–12% Bis-Tris-PAGE (Invitrogen, Carlsbad, CA, USA) under reducing conditions and transferred to PVDF membranes (Millipore, Billerica, MA, USA). After transfer, membranes were blocked with 5% milk in PBS, 0.1% Tween-20 for 1 h at room temperature and incubated with primary antibodies for anti-β-actin (1:5000), anti-gasdermin (1:1000), anti-opsin mAb 4D2 (1:1000) overnight at 4 °C. Membranes were washed and incubated with goat anti-rabbit (1:3000) or goat anti-mouse (1:3000) horseradish peroxidase-conjugated secondary antibodies for 1 h at room temperature. The blots were developed using ECL (SuperSignal^®^ West Dura extended duration substrate (Thermo Scientific, Waltham, MA, USA) and captured on Li-Cor Odyssey Fc image reader and quantified as described [14].

### 2.8. Staining for Neutral Lipids

BODIPY™493/503 (4,4-Difluoro-1,3,5,7,8-Pentamethyl-4-Bora-3a,4a-Diaza-s-Indacene, Molecular Probes, Eugene, OR, USA; D3922) was used to visualize neutral lipid-rich deposits. BODIPY™493/503 stock solution (0.5 mg/mL) was prepared in 100% ethanol and diluted in 1xPBS to 10 μg/mL before the experiment. Cells (RPE and RPE^-LC3B^) were incubated with OS as described above. Cells were fixed with 4% PFA for 10 min at RT, washed in PBS, incubated in BODIPY^TM^493/503 for 1 h at RT in the dark, followed by nuclear staining (Hoechst 33298) and three, 5-min PBS washes. Images were captured on a Nikon A1R laser scanning confocal microscope with a 60X water objective at 18 °C, and the data were analyzed using Nikon Elements AR 4.30.01 software [27]. Briefly, after background subtraction, sum intensity for the BODIPY™493/503 staining was obtained for each image. The intensity measure for each field was normalized relative to the number of cells in the field and averaged (*n* = 3).

### 2.9. Staining for Mitochondria 

Mitochondria were visualized using MitoTracker™ Red CMXRos (Invitrogen M7512, Carlsbad, CA, USA). Cells (RPE and RPE^-LC3B^) grown on glass-bottom dishes were pretreated with 100 µM LGM for 30 min as described above. The cells were rinsed in serum-free media followed by incubation with 400 nM MitoTracker™ Red CMXRos diluted in serum-free medium at 37 °C for 15 min, essentially as described [35,53]. The cells were washed in serum-free media (3 × 5 min) and fixed in 4% paraformaldehyde for 10 min. After Hoechst33298 staining and 3 successive PBS washes, the cells were imaged on a Nikon A1R laser scanning confocal microscope with a 100X oil objective at 18 °C, and the data were analyzed using Nikon Elements AR 4.30.01 software. Briefly, after background subtraction, sum intensity was obtained for each image and the intensity measure for each field was normalized to the number of cells in the field and averaged (*n* = 3).

### 2.10. Staining for Actin 

For actin filament staining, cells were fixed in 4% PFA, washed, permeabilized and blocked in blocking solution containing 5% BSA and 0.2% Triton X-100 in PBS (PBST) at 37 °C for 1 h, incubated with the Alexa Fluor 594 Phalloidin (1 Unit/200 µL) (Invitrogen, Carlsbad, CA, USA, A12381) o/n at 4 °C, washed in PBST and imaged using a Nikon A1R laser scanning confocal microscope with a PLAN APO VC 60× water (NA 1.2) objective at 18 °C

### 2.11. Transepithelial Resistance (TER)

Transepithelial electrical resistance (TER) of RPE and RPE^-LC3B^ grown on 12-well transwell inserts (12 mm diameter, 0.4 μm pore size, Corning, UK) were measured as an indicator of barrier integrity/polarisation using an EVOM2 epithelial voltohmmeter and 4 mm STX2 chopstick electrode per the manufacturer’s instructions (World Precision Instruments Inc., Sarasota, FL, USA). After the electrode was sterilized in 70% ethanol, rinsed in ddH20 and equilibrated in prewarmed culture medium, it was simultaneously introduced into both chambers. TER measurements from at least three cultures per condition, no addition (NA), +LGM2605 (100 µM), +OS and +OS and LGM2605 (100 µM) were obtained over a period of 7 days. Measurements were made every other day. All measurements were performed within 6 min at room temperature after removal from the incubator.

### 2.12. TUNEL Staining

For Tunel staining of cells, ApoTag in situ apoptosis kit (Chemicon, Miyagi, Japan) as we have described previously [54]. Briefly, 4% PFA fixed cells were post-fixed in pre-chilled ethanol: acetic acid (2:1) for 5 min at −20 °C, washed, treated with proteinase K (20 ug/mL) for 5 min at RT. The cells were then incubated in equilibration buffer, treated with terminal deoxynucleotidyl transferase (TdT) enzyme at 37 °C for 1h, incubated with anti-digoxigenin fluorescein conjugate (1:1) at RT for 30 min, followed by nuclear staining (Hoechst 33258) and imaging.

### 2.13. Ketone Body Measurements, β-Hydroxybutyrate (β-HB) Assay

Preparation of metabolic substrates—Ringer’s solution was prepared using the basic chemical components of RPE cell culture medium: CaCl_2_ (1.1 mM), KCl (4.2 mM), NaCl (120.6 mM), NaHCO_3_ (14.3 mM), MgCl_2_ (0.3 mM) and HEPES (15 mM). HEPES was dissolved separately and titrated to pH 7.4 with *N*-methyl-D-glucamine. L-Carnitine (1 mM) in Ringer’s solution was added immediately before each experiment; the solution was equilibrated to pH 7.4 with CO_2_ and filter-sterilized. Photoreceptor OSs purified from frozen dark-adapted bovine retinas were added to cultured RPE cells as indicated above. The phospholipid content of the OS was determined using a Malachite Green assay kit per the manufacturer’s instructions (K-1500; Echelon Biosciences, Salt Lake City, UT, USA) as originally described in (46). On the day of the experiment, the OSs were thawed, pelleted and resuspended in Ringer’s solution at a final concentration of 200 µM (based on total phospholipid content). Ringer’s solution containing different substrates was added to the apical chamber of RPE, RPE^-MREG^ and RPE^-LC3B^ cells grown on 12-well Transwell filters. Ringer’s solution (115 µL) was collected from the apical and basal chamber and analyzed for β-HB at the 2 and 3 h time points essentially using the β-hydroxybutyrate LiquiColor kit (Stanbio, Boerne, TX, USA; catalog no. 2440-058) as described previously [14]. No βHB was detected with OS alone. In studies utilizing LGM2605, the cells were preincubated for 30 min with 100 µM LGM2605.

### 2.14. Lactate Dehydrogenase (LDH) Assay

LDH released into the extracellular solution was measured as an indicator of cell membrane integrity using a coupled reaction where tetrazolium salt was reduced to the colored product formazan by enzyme activity (cytotoxicity detection kit LDH; Roche Applied Science, Mannheim, Germany) as we have described previously [54,55]. The solution itself did not affect the LDH assay.

### 2.15. Lipid Peroxidation

RPE lysates were isolated, as described above, at the indicated time points and immediately prepared for 4-HNE analysis. Cleared RPE lysates obtained by centrifugation at 2000× *g* for 3 min were used in twofold series dilutions. Samples were analyzed using 4-HNE-ELISA kit from Cell Biolabs, San Diego, CA, United States according to the manufacturer’s directions using a Multiskan MCC plate reader (Thermo Fisher Scientific, Waltham, MA, United States) [27]. Protein was quantified using Bradford reagent (Thermo Fisher, Waltham, MA, USA).

### 2.16. Cytokine Analysis

Cytokine production was measured in the culture supernatants from RPE and RPE^-LC3B^ cells challenged as described above in the presence or absence of LGM2605 at the concentrations indicated in the Figure legends. Culture supernatants collected at the time points indicated in the figure legends were analyzed by ELISA for IL-1 β (Quantikine Elisa Kit; R&D Systems) and IL-18 (Quantikine Elisa kit; R&D Systems, #DL180) commercially available kits according to the manufacturer’s instructions [55]. In each instance, the amount of cytokine present in the supernatant was determined using a standard curve.

### 2.17. Statistical Analyses

We used a two-way analysis of variance (ANOVA) to evaluate the effect of the type of RPE cell (RPE, RPE^-LC3B^) and the incubation condition (NA, OS, LGM2605, OS + LGM2605). The ANOVA model included the two main effects from type of RPE cell and the incubation condition and their interaction term. If there was no significant interaction (i.e., *p* > 0.05) between RPE cell type and the incubation condition, two-way ANOVA was performed without the interaction term, and the main effects for the type of RPE cell and the incubation condition were determined. If there was significant interaction (*p* < 0.05) between RPE cell type and the incubation condition, the main effect for the type of RPE cell and the incubation condition was not determined. Instead, the pairwise comparisons across all the combinations of RPE cell type and incubation condition were performed, and corrections for multiple comparisons were made using Holm–Sidak method. When appropriate, the unpaired *t*-test was also used to compare two means and a 2-tailed value of *p* < 0.05 was considered statistically significant. * indicates *p* < 0.050, ** indicates, *p* < 0.020 and *** indicates *p* < 0.001.

## 3. Results

### 3.1. Lipid Overload and Cytokine Release in RPE 

**Figure 1:**
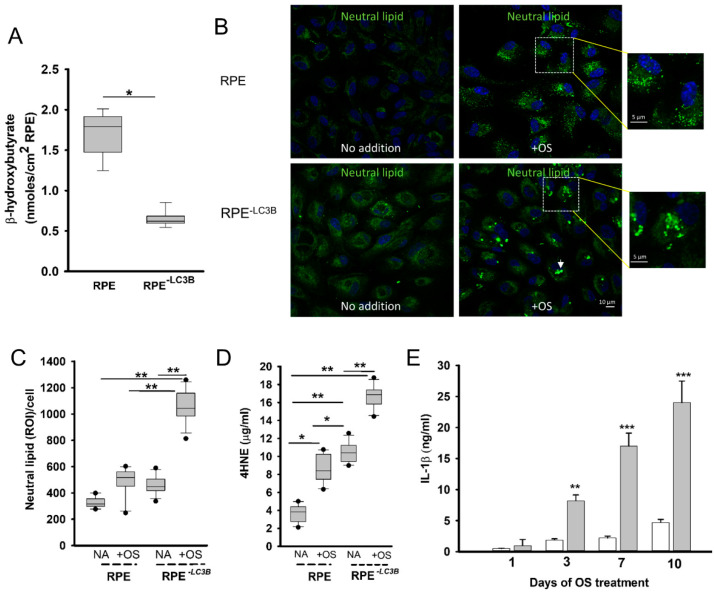
Loss of LC3B-associated OS phagocytosis results in lipid overload, peroxidation and cytokine release (**A**–**E**). (**A**). RPE or RPE^-LC3B^ cells were incubated in the chamber with OSs and the apical supernatant was evaluated for β HB content after 2 h. The box plots show data for three independent experiments, each done in triplicate. One-way ANOVA was used for comparison across the RPE cells, and post-hoc pairwise comparison using Tukey correction was used. * indicates *p* < 0.050. (**B**). RPE or RPE^-LC3B^ cells were incubated with OS once daily for 7 days. Representative confocal image of neutral lipid deposits (green) detected by staining with BODIPY493/503 and nuclei (blue) stained with Hoechst 33342. White arrowhead indicates large aggregates on the RPE^-LC3B^. (**C**). The intensity of BODIPY493/503 staining was quantified and puncta localized as indicated. INSETS: depict the size and general shape of lipid deposits. The box plots show data for three independent experiments, each done in triplicate. One-way ANOVA was used for comparison across the conditions tested, and post-hoc pairwise comparison using Holm–Sidak correction was used. For each independent experiment, we analyzed 5 fields, with each field containing at least 25 cells. ** indicates *p* < 0.020. (**D**). The level of 4-hydroxynonenol (4-HNE) protein adducts measured by ELISA for RPE or RPE^-LC3B^ cells fed OS in the long-term challenge study. The box plots show data for three independent experiments, each done in triplicate. One-way ANOVA was used for comparison across the conditions tested, and post-hoc pairwise comparison using Holm–Sidak. * indicates *p* < 0.050 and ** indicates, *p* < 0.020. (**E**). Levels of IL-1 β released into apical media by ELISA for RPE (white bars) or RPE^-LC3B^ cells (grey bars) challenged with OS in long-term fed study. Each of three samples was tested in triplicate per cell type on each day indicated. Bar plots show mean ± SEM. Data were analyzed using unpaired Student’s *t*-test with *p* values as indicated. ** *p* < 0.002, and *** *p* < 0.001. *p* values represent a comparison between RPE (white bar) and RPE^-LC3B^ (gray bar) on the days indicated.

We first sought to determine if we could recapitulate aspects of the *Lc3b^-/-^* RPE phenotype in vitro, with specific emphasis on lipid-mediated stress. We asked if delayed phagosome degradation correlated with decreased ketogenic efficiency in RPE^-LC3B^ cells. In RPE^-LC3B^ cells, β- hydroxybutyrate (β-HB) release decreased by 63% as compared to RPE controls (Figure 1A).

In the next series of studies, we used a long-term, 7 day OS challenge model in which we could assess the cumulative effects of OS uptake and ketogenic insufficiency over time. On day 7 of the OS challenge, RPE^-LC3B^ cells accumulated twice as much intracellular lipid compared to control RPE (Figure 1B,C). The lipid deposits in RPE^-LC3B^ appeared as larger aggregates (Figure 1B, arrowhead and inset). When total fluorescence intensity of regions of interest/cell (ROI/cell) was compared, a statistically significant increase in neutral lipid-associated fluorescence was observed in RPE^-LC3B^ challenged with outer segments versus no addition of OS, as well as RPE + OS (Figure 1C). Enhanced lipid accumulation in the oxidative stress environment of the RPE is predicted to result in enhanced lipid peroxidation. Lipid peroxidation levels were determined as 4-hydroxynonenal (4-HNE)-adducts by ELISA. 4-HNE peroxidation products were 3 fold higher in the RPE^-LC3B^ cells than controls at day 7 of OS challenge (Figure 1D). By day 7, IL-1β release was 5- fold higher in RPE^-LC3B^ cells as compared to controls (Figure 1E). Membrane pores associated with the release of IL-1 β may be due to the targeted cleavage of gasdermin [56]. Concomitant with lipid deposits in RPE^-LC3B^ was the cleavage of gasdermin (GSDMD), resulting in the formation of an N-terminal 30 kda fragment (GSDMD-N) shown to induce membrane pores (Supplementary Figure S1) [55].

### 3.2. LGM2605 Restores Oxidative Capacity and Decrease Cytokine Release in RPE

We then tested the hypothesis that LGM2605, the synthetic lignin, secoisolariciresinol diglucoside (SDG) protects against oxidative stress and restores metabolic capacity to RPE under lipid overload.

**Figure 2:**
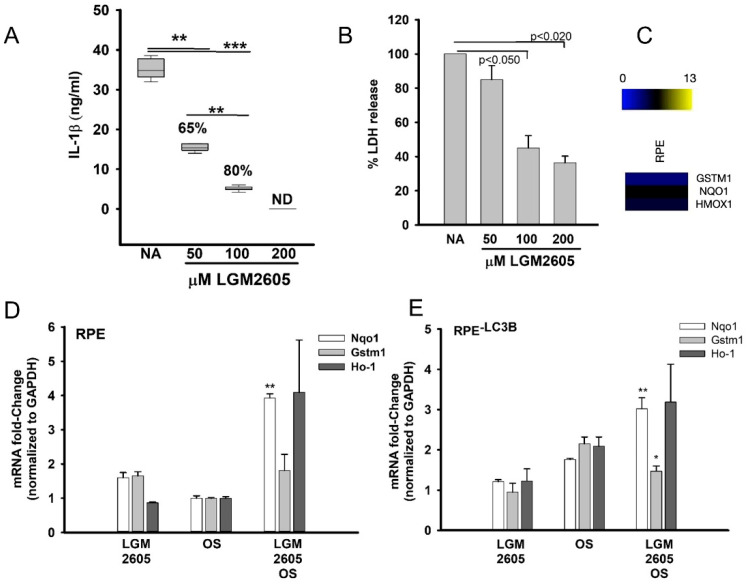
LGM2605 decreases oxidative stress-mediated cytokine release and upregulates Nrf-2 dependent gene expression (**A**–**E**). (**A**) Levels of IL-1β released into apical media by ELISA for RPE or RPE^-LC3B^ cells stressed with 200 µM H_2_O_2_ and 10 ng/mL TNF-α. Cells were pretreated with LGM2605 (50–200 µM). The box plots show data from three independent experiments, each done in triplicate. One-way ANOVA was used for comparison across increasing concentrations of LGM2605, and post-hoc pairwise comparison using Holm–Sidak correction was used. ** indicates *p* < 0.020, *** indicates *p* < 0.001. (**B**) Levels of LDH by ELISA for RPE or RPE^-LC3B^ cells stressed with 200 µM H_2_O_2_ and 10 ng/mL TNF-α. Cells were pretreated with LGM2605 (50–200 µM) *n* = 3, Data are shown as mean ± SEM, of three independent experiments each in duplicate. (**C**) Heatmap analysis of publicly available microarray datasets for RPE (GSE62947) identified three genes of moderate abundance in RPE, NQO1, GSTM1 and HO-1 M. (**D**) Fold-change in mRNA of Nrf-2 dependent genes, Npo1, Gstm and Ho-1 in RPE cells with our without OS challenge or LGM2605 pretreatment. Data were shown as mean ± SEM and analyzed using Student’s *t*-test. ** indicates *p* < 0.020. (**E**) Fold-change in mRNA of Nrf-2 dependent genes, Npo1, Gstm and HO-1 in RPE^-LC3B^ cells with our without OS challenge or LGM2605 pretreatment. Data were shown as mean ± SEM and analyzed using Student’s *t*-test. * indicates *p* < 0.050, ** indicates *p* < 0.020.

In this first series of experiments, we sought to determine if oxidative stress triggered cytokine release was alleviated by LGM2605 using a well characterized oxidative stress model [47,48]. RPE and RPE^-LC3B^ cells treated with 200 µM H_2_0_2_ and 10 ng/mL TNF-α and challenged with OS were pretreated with varying concentrations of LGM2605 (50–200 μM). IL-1β release and cytotoxicity was analyzed 24 h later. IL-1β release decreased by 65% with 50 µM LGM2605, with over 80% decrease when LGM2605 was increased to 100 µM (Figure 2A). Coincident with the decrease in IL-1β was a decrease in stress-induced cytotoxicity, with a 60% decrease in cell leakiness to LDH in the presence of 100 µM LGM2605 (Figure 2B). Neither TNF-α or H_2_O_2_ alone triggered a significant effect under these conditions; IL-1β release with 600 µM H_2_O_2_ was 0.28 ± 0.08 ng/mL and with only 10 ng/mL TNF-α, IL-1β release was 0.34 ± 0.52 ng/mL, consistent with observations by other groups [48]. LGM2605 at the concentrations used herein did not alter RPE barrier integrity as measured by transepithelial resistance (TER). Routinely, TER values were between 156 and 164 (Ω/cm^2^) ± LGM2605 (50–100 µM) and are presented in Supplementary Figure S2A. RPE cells remain viable as, the addition of LGM2605 did not alter RPE cell morphology (Supplementary Figure S2B) and did not result in RPE cell death as detected by TUNEL staining (Supplementary Figure S3). Routinely, over 98% of the cells treated with LGM2605 (50 µM to 200 µM) were TUNEL negative. Moreover, LGM2605 addition did not affect phagocytic function per se, as opsin levels 2 h after OS ingestion were equal in the presence or absence of LGM2605 (Supplementary Figure S2C). Overall, LGM2605 decreased IL-1β secretion and protected the cells from oxidative stress-induced cytotoxicity.

Using this LGM2605 dose–response data, we turned our attention to understanding if and how LGM2605 exerts an effect on our lipid overload stress model. Analysis of publicly available microarray datasets for RPE (GSE62947) identified three genes of moderate abundance in RPE, *NQO1*, *GSTM1* and *HO-1* (Figure 2C). The expression of these genes in RPE and RPE^-LC3B^ was analyzed in the presence of 100 µM LGM2605 in our 7 day lipid overload stress model (Figure 2D,E). We detected a significant increase in *NQO1* in RPE (*p* = 0.0011) and RPE^-LC3B^ (*p* = 0.0238) cells treated with OS plus LGM2605 (Figure 2D,E) as compared to just OS (3.93 and 3.02 fold increase, respectively).

**Figure 3:**
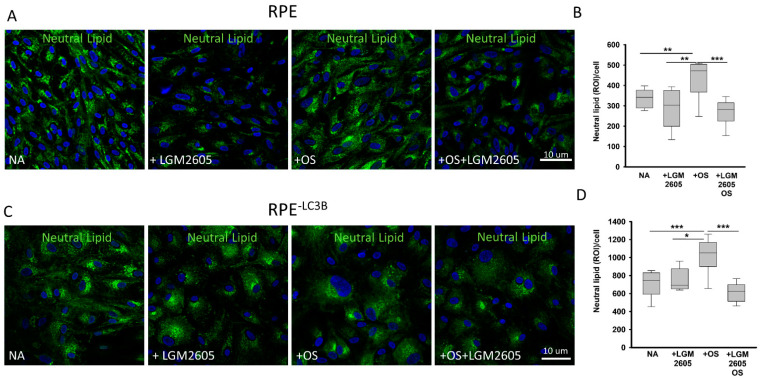
LGM2605 diminishes intracellular neutral lipid accumulation and peroxidation (**A**–**D**). (**A**) Representative confocal image of neutral lipid deposits (green) detected by staining with BODIPY493/503 and nuclei (blue) stained with Hoechst33342. RPE cells at day 7. Each day of a 7- day long study, cells were either left untreated, pretreated with LGM2605 (100 µM), or challenged with OS, with or without (LGM2605 (100 µM) pretreatment. (**B**) The intensity of BODIPY493/503 staining was quantified and puncta localized as indicated. Plots show data for three independent experiments, each done in triplicate. Two-way ANOVA was used for comparison across the conditions tested, and post-hoc pairwise comparison using Holm-Sidak correction was used. For each independent experiment, we analyzed 5- fields of cells each containing at least 20 cells. ** indicates, *p* < 0.020 and *** indicates *p* < 0.001. (**C**) Representative confocal image of neutral lipid deposits (green) detected by staining with BODIPY493/503 and nuclei (blue) stained with Hoechst33342 in RPE^-LC3B^ cells at day 7. Cells were either left untreated, treated with LGM2605 (100 µM), OS, or LGM2605 (100 µM) + OS. (**D**) The intensity of BODIPY493/503 staining was quantified and puncta localized as indicated. Plots show data for three independent experiments, each done in triplicate. Two-way ANOVA was used for comparison across the conditions tested, and post-hoc pairwise comparison using Holm–Sidak correction was used. For each independent experiment, we analyzed 5 fields of cells, each containing at least 20 cells. * indicates *p* < 0.050. *** indicates *p* < 0.001.

The RPE^-LC3B^ cell, and to a lesser extent RPE, showed elevated levels of BODIPY staining indicative of neutral lipid accumulation after daily challenge with OS over 7 days (Figure 3B,D). When total fluorescence intensity of regions of interest/cell was compared, a statistically significant decrease was observed when cells were pretreated with LGM2605 (100 µM, 30 min) prior to daily OS challenge in both RPE^-LC3B^ (Figure 3C,D; lower panel) and to a lesser extent in control RPE (Figure 3A,B; top panel).

In Supplementary Figure S4, a weak green autofluorescence is noticeable only under enhanced settings. This intrinsic fluorescence is not influenced by LGM2605. The source of autofluorescence in APRE-19 cells are lipofuscin fluorophores, such as A2E, *N*-retinylidene-*N*-retinyl-ethanolamine (Em λ_max_ 570 nm) [10,34,55,56]. Its fluorescence signal is negligible and does not influence the cell imaging experiments using very bright fluorophores BODIPY™493/503 (Em λ_max_ 503 nm), Hoechst 33298 (Em λ_max_ 460 nm) or MitoTracker™ Red CMXRos (Em λ_max_ 644 nm). It should be noted that LGM2605 does not possess quenching properties for any of the above fluorophores, because it does not absorb light in the range of 400–800 nm (Supplementary Figure S4B).

**Figure 4:**
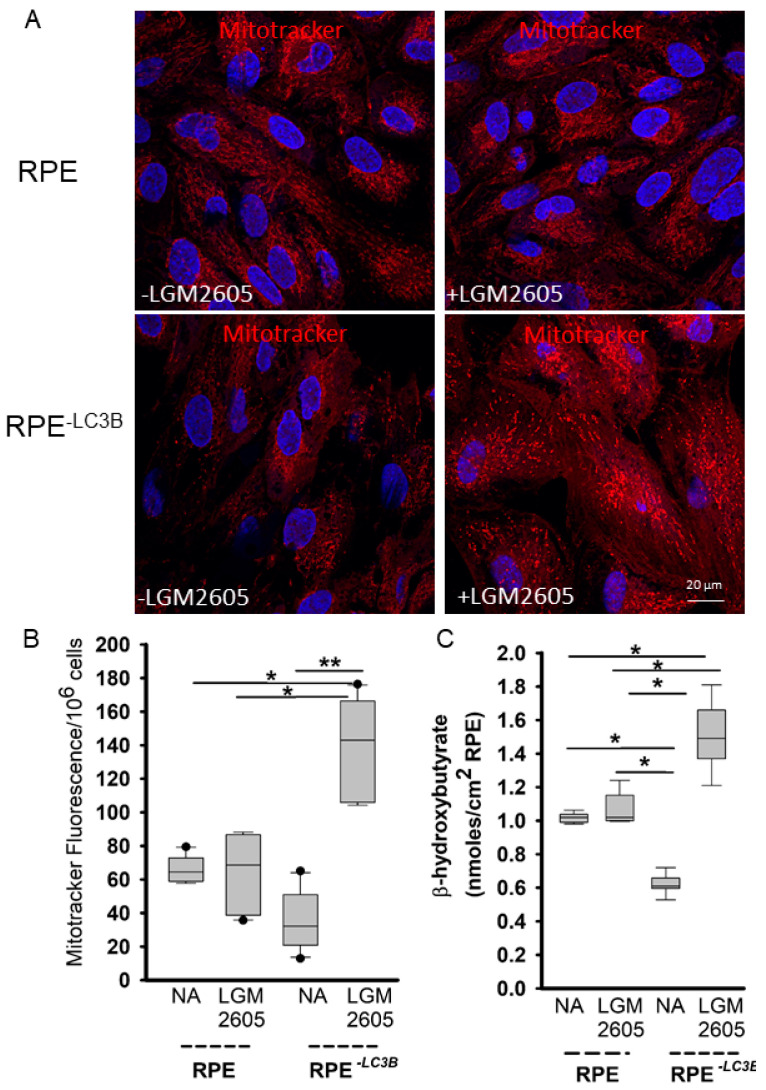
LGM2605 corrects lipid overload mediated mitochondrial stress (**A**–**C**). (**A**) Representative confocal image of mitochondria (red) detected by staining with Mitotracker Red and nuclei (blue) stained with Hoechst 33342 of RPE and RPE^-LC3B^ cells at day 7 of OS treatment. 100 µM LGM2605 was added in +LGM2605. (**B**) Mitotracker fluorescence was quantified. Plots show data for three independent experiments, each done in triplicate. Two-way ANOVA was used for comparison across the conditions tested, and post-hoc pairwise comparison using Holm–Sidak correction was used. For each independent experiment, we analyzed 5 fields of cells at 60X magnification. * indicates *p* < 0.050, ** indicates, *p* < 0.020. (**C**) RPE or RPE^-LC3B^ cells were pretreated with 100 µM LGM2605 and subsequently incubated with OSs and the apical supernatant was evaluated for β HB content after 2 h. Plots show data for three independent experiments, each done in triplicate. Two-way ANOVA was used for comparison across the conditions tested, and post-hoc pairwise comparison using Holm–Sidak correction was used. * indicates *p* < 0.050.

**Figure 5:**
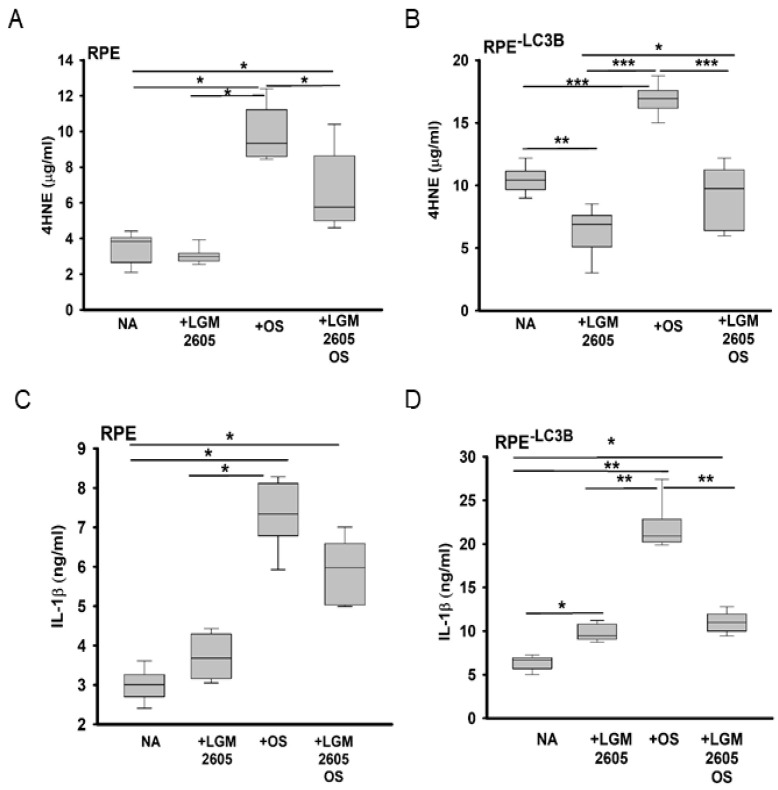
LGM2605 acts as an antioxidant and anti-inflammatory agent in a model of lipid dysregulation (**A**–**D**). (**A**) Level of 4-hydroxynonenol protein adducts measured by ELISA for RPE cells at day 7 of 7-day OS feed. Cells were either left untreated, treated with LGM2605 (100 µM), OS or LGM2605 (100 µM) + OS. (**B**) Levels of 4-hydroxynonenol protein adducts measured by ELISA for RPE^-LC3B^ cells at day 7 of 7-day OS feed. Cells were either left untreated, treated with LGM2605 (100 µM), OS or LGM2605 (100 µM) + OS. (**C**) Levels of IL-1β released into apical media by ELISA for RPE cells at day 7 of 7-day OS feed. Cells were either left untreated, treated with LGM2605 (100 µM), OS or LGM2605 (100 µM) + OS. (**D**) Levels of IL-1β released into apical media by ELISA for RPE^-LC3B^ cells at day 7 of 7-day OS feed. Cells were either left untreated, treated with LGM2605 (100 µM), OS, or LGM2605 (100 µM) + OS. All plots show data for three independent experiments, each done in triplicate. Two-way ANOVA was used for comparison across the conditions tested, and post-hoc pairwise comparison using Holm–Sidak correction was used. * indicates *p* < 0.050, ** indicates, *p* < 0.020 and *** indicates *p* < 0.001.

Based on the similarity between RPE and cardiomyocyte metabolic capacity, we assessed whether the beneficial effects of LGM2605 involved changes to mitochondrial function. RPE and RPE^-LC3B^ cells treated with LGM2605 daily as described above showed an increase in MitoTracker red staining, suggestive of enhanced mitochondrial abundance in RPE^-LC3B^ (Figure 4A,B). In accordance with these results, ketogenesis measured as β-HB release reflecting mitochondrial function also increased in LGM2605 treated samples (Figure 4C). Given that LGM2605 restored ketogenic capacity, thereby reducing intracellular lipid deposition, we determined if there was a corresponding change in 4-HNE-peroxidation adducts. LGM2605 treatment decreased 4-HNE levels in RPE^-LC3B^ cells with or without OS challenge but had no effect on the minimal amount of 4-HNE in RPE controls (Figure 5A,B). Decreases in the proinflammatory 4-HNE adducts also contributed to a decrease in IL-1β secretion in LGM2605 treated RPE^-LC3B^ cells after OS challenge, with a trend towards diminished IL-1β in RPE controls (Figure 5C,D).

In contrast to the well-documented release of IL-1β in response to RPE para-inflammation there is considerable debate regarding whether IL-18 contributes to or protects from AMD-associated phenotypes including choroidal neovascularization (CNV) [57,58,59,60]. IL-18 is a multifunctional cytokine [61]; IL-18 offers protection against colitis, with a deficiency in IL-18 proposed to predispose the host to inflammation associated with colitis and epithelial damage [62,63,64,65]. Given the importance of this cytokine in understanding RPE pathophysiology, we asked if LGM2605 treatment modulated IL-18 release. IL-18 doubled upon OS challenge in both RPE and RPE^-LC3B^ cells within the first two days. LGM2605 contributed to a doubling of IL-18 release by day 6 in RPE and by day 2 in the RPE^-LC3B^ (Table 1).

## 4. Discussion

In the present study, we focused on defining the relationship between defective phagosome clearance and loss of lipid homeostasis resulting in oxidative stress and cytokine release by the RPE. We further evaluated the potential for LGM2605, a synthetic lignin with potent antioxidant and mito-protective properties, to restore RPE lipid homeostasis and ameliorate cytokine release. In vivo, delayed phagosome maturation, specifically the inability to utilize LC3B associated phagocytosis (LAP), resulted in diminished chromophore recycling of a process necessary to support visual function [66] as well as loss of protective lipid mediators [48,67,68]. These consequences, as well as lipid accumulation and peroxidation, all contributed to an overall proinflammatory environment resulting in microglial infiltration and loss of visual function [27]. Herein, we asked if lipid dysregulation contributes to oxidative stress and inflammation using RPE^-LC3B^ cells in which the major trafficking partner in LAP, LC3B, has been knocked down. We assessed the consequences of defective metabolic capacity in a cumulative OS uptake model [49], designed to recapitulate the daily ingestion and degradation of lipid-rich OS carried out by the RPE in vivo. This model has been used previously to assess the effectiveness of dopamine receptor agonists on decreasing lipofuscin-like autofluorescent accumulation in RPE [49]. RPE^-LC3B^ cells show an ~65% decrease 5 in fatty acid oxidation (Figure 1A) and a concomitant increase in intracellular lipid deposits (Figure 1B,C), which serve as substrates for oxidation reactions resulting in the generation of 4-HNE. 4-HNE levels in RPE^-LC3B^ were ~50% higher than control RPE (Figure 1D). Oxidized lipids often directly contribute to tissue injury or react with amines on proteins to form oxidation specific epitopes, which can induce an innate immune response. Many oxidation-dependent changes, including, 4-HNE adducts have been identified in AMD, further suggesting that oxidative stress is an important factor in disease development. Over the 7 day OS uptake period, a steady time-dependent increase in IL-1β release was seen in RPE^-LC3B^ while little IL-1 β was released from control cells. Interestingly, we observed a lipid accumulation-based increase in gasdermin processing (Supplementary Figure S1). One of the key steps in caspase-1 and -4-mediated activation of pyroptosis and the resulting release of proinflammatory cytokines is the cleavage of GSDMD. Caspase-1 and caspase-4 cleavage removed the C-terminal fragment; this releases and activates the N-terminal fragment, GSDMD-NT. This fragment is directly responsible for pore formation in the plasma membrane, which in turn enhances cytokine release and ultimately leads to cell death, as indicated by LDH release [67]. Gasdermin D levels have been shown to be elevated in the RPE in human eyes with geographic atrophy [68]. In this regard, studies looking at the relationship between gasdermin pore formation mediated IL-1 β release and lipid homeostasis are underway. Collectively, these in vitro studies establish a model in which one can directly assess the impact of lipid metabolic dysregulation on inflammation. Moreover, they also provide a platform for us to assess the effectiveness of LGM2650, a chemically synthesized secoisolariciresinol diglucoside (SDG).

SDG is a bioactive lignan component of flaxseed, is a non-toxic whole grain that consists of high concentrations of omega-3 fatty acids and lignans. SDG is a potent antioxidant with anti-inflammatory and antifibrotic properties; the beneficial effects of SDG have been documented in treating hypercholesterolemia, diabetes, postmenopausal symptoms, cardiovascular disease, metabolic syndrome, bone disease, ischemia-reperfusion injury, radiation-induced pneumonopathy and hyperoxia [69], and references therein. Chemically synthesized SDG, LGM2605 is effective in several preclinical models of disease in which oxidative stress and inflammation play a prominent role in pathogenesis [35,39,40,41]. Herein, we show for the first time that LGM2605 protects the RPE against lipid overload and oxidative stress mediated cytokine release. In addition, we show LGM2605’s mitoprotective properties in increasing mitochondrial abundance and mitochondrial metabolic capacity with enhanced FAO and ketogenesis.

LGM2605 protected RPE cells from H_2_O_2_ induced oxidative damage in a dose-dependent manner (0–200 µM); when RPE was pretreated with LGM2605, prior to stress induction, an 80% decrease in IL-1β release was observed with a corresponding increase in cell viability (Figure 2A,B). These results are suggestive of the cytoprotective properties of quercetin, a flavonoid compound that, among other properties, activates Nrf2 dependent gene expression to protect RPE [47]. Similarly, LGM2605 stimulates Nrf2 dependent gene expression, and herein, we show a statistically significant increase in Nqo-1 and HO-1, when RPE cells were pretreated with LGM2605 prior to OS addition (Figure 2D). In cells, similar increases in Nqo-1 and Ho-1 were observed upon LGM2605 treatment (Figure 2E), albeit the RPE^-LC3B^ baseline levels of these genes were higher in cells treated with OS only, suggesting that in the absence of lipid degradation, there is a compensatory upregulation of stress-response genes.

Although the exact mechanisms of action of SDG have not been fully explored, it has known protective mechanisms including direct free radical scavenging activity, induction of antioxidant response, anti-inflammatory properties, repression of inflammatory mediators and mitochondrial homeostasis. The studies shown in Figure 3 also suggest antisteatosis. After 7 days of daily OS challenge we observed a significant increase in neutral lipids accumulated in both RPE and RPE-LC3B cells. When cells were pretreated with 100 µM LGM2605 daily prior to OS addition, a substantial decrease in neutral lipid deposits was observed; levels of intracellular neutral lipid fell to levels found in untreated cells. LGM2605 had no effect on phagosome maturation as detected by opsin degradation (Supplementary Figure S2C).

The most straightforward hypothesis to consider in assessing the molecular mechanism by which LGM2605 decreased lipid accumulation was to ask whether this compound was able to enhance lipid degradation, thereby decreasing available lipid for deposition. Changes in metabolites of mitochondrial metabolism and glucose utilization are most commonly associated with the aging retina [70] metabolic dysfunction, including decreased energy metabolism and impaired antioxidant defense, has been reported in retina and RPE [71]. Age-related retinal disease is proposed to involve a cumulative metabolic decompensation in which the loss of mitochondrial oxidative function and mitochondrial stress contribute to pathophysiology [1,2]. Therefore, to assess whether the beneficial effects of LGM2605 involves changes in mitochondrial numbers, we stained 7 day OS challenged RPE and RPE^-LC3B^ cells with Mitotracker red at day 7. While there was a trend towards increase Mitotracker staining, and hence mitochondrial numbers, in the RPE with LGM2605 treatment in the RPE-LC3B, a 2.5 fold statistically significant increase in mitochondrial numbers was observed (Figure 4A,B). Consistent with enhanced mitochondrial function is the stimulation of ketogenesis with twice as much βHB released in RPE-LC3B treated with 100 µM LGM2605. Similarly, enhanced ketogenic capacity ameliorates aspects of hepatic steatosis [72]. Clues to the relationship between LGM2605 and mitochondrial function come from the observations that Nrf-2 affects mitochondrial membrane potential, fatty acid oxidation, as well as substrate availability for respiration and ATP production [73,74]. Thus, it is tempting to suggest that LGM2605 mediated Nrf-2 dependent gene expression includes upregulation of genes involved in fatty acid oxidation and mitochondrial respiration. Albeit, in cardiac myocytes, LGM2605 alleviated mitochondrial oxidative stress without altering fatty acid and glucose metabolism-related genes, whether the same is true in RPE cells is presently under investigation [35]. Treatment with LGM2605 not only restored metabolic efficiency but, by doing so, also contributed to decreased lipid peroxidation and associated cytokine release (Figure 5A–D).

**Figure 6:**
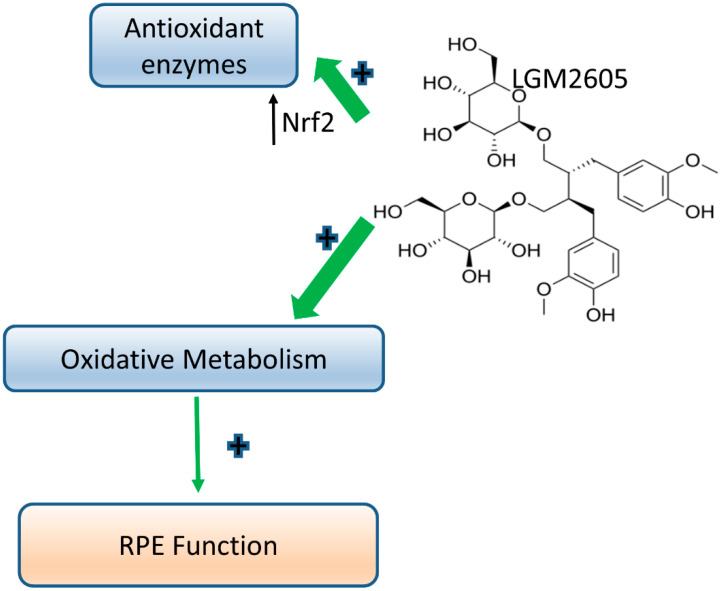
Schematic representation of LGM2605′s beneficial properties in restoring oxidative metabolism and stimulating antioxidant and anti-inflammatory processes in the RPE.

These studies expand on numerous models, including those from our group that demonstrated that impaired phagosome clearance contributes to cumulative oxidative stress manifest as the formation of oxidative lipid and DNA adducts, cytokine release and para-inflammation, resulting in loss of function [1,2,9,73]. The causal link between altered lipid homeostasis and chronic low-grade inflammation is not limited to the retina. Nonalcoholic fatty liver disease (NAFLD) and nonalcoholic steatohepatitis provide examples in which lipid overload combined with ketogenic insufficiency is manifest as hepato-inflammation [72,75]. Similarly, cardio myocytes which predominantly use fatty acids as an energy source, exhibit cumulative metabolic decompensation and inflammation under infection-induced stress [35]. Both hepatocyte and cardio myocyte pathophysiology in NAFLD and heart failure, respectively, are similar to changes observed in RPE in age-related diseases [2,72,73,74,75,76]. LGM2605 restores oxidative capacity and aids in promoting an antioxidant anti-inflammatory environment to maintain RPE health (Figure 6) similar to cardiomyocytes [35]. Our observations are of particular significance in considering LGM2605 as a potential therapeutic, as LGM2605 crosses the blood–brain barrier and is neuroprotective [39]; its role in retinal homeostasis is just beginning to be elucidated. Our own future studies are focused on establishing an efficacious mode of LGM2650 delivery to the back of the eye. We predict LGM2605 may serve to protect the retinal Burch’s membrane and choroidal vasculature from extraneous lipid debris accumulation, thereby maintaining visual function in aging and age-related disease

## Figures and Tables

**Table 1:** IL-18 released into apical supernatant. RPE or RPE^-LC3B^ cells were incubated once daily for 7 days. Each day of a 7-day long study, cells were either left untreated, pretreated with LGM2605 (100 µM) or challenged with OS, with or without (LGM2605 (100 µM) pretreatment. IL-18 released into the apical media as measured by ELISA at day 2 and day 6 is indicated. Data shown is the mean ± SEM of three independent experiments, each in duplicate. * *p* < 0.050, and ** *p* < 0.020.

SAMPLE	IL-18 Released ng/mL			
	NA	+LGM2605	+OS	+OS and LGM2605
**RPE**				
**Day 2**	0.11 ± 0.01	0.73 ± 0.87	1.47 ± 0.18 *	2.21 ± 0.12 **
**Day 6**	0.48 ± 0.04	0.98 ± 0.12	0.73 ± 0.84	6.04 ± 0.44 **
**RPE^-LC3B^**				
**Day 2**	0.17 ± 0.02	1.47 ± 0.12 *	2.09 ± 0.33 **	4.56 ± 0.89 **
**Day 6**	0.11 ± 0.01	0.36 ± 0.44	1.10 ± 0.0.81 *	0.73 ± 0.09 *

## Supplementary Materials

The following are available online at https://www.mdpi.com/article/10.3390/ijms22115764/s1.
