# Supplementary figures and images for: Assessment of a Small Molecule Synthetic Lignan in Enhancing Oxidative Balance and Decreasing Lipid Accumulation in Human Retinal Pigment Epithelia
Source: Int J Mol Sci. 2021 May 28;22(11):5764. doi: 10.3390/ijms22115764 (PMC8198017; doi:10.3390/ijms22115764)

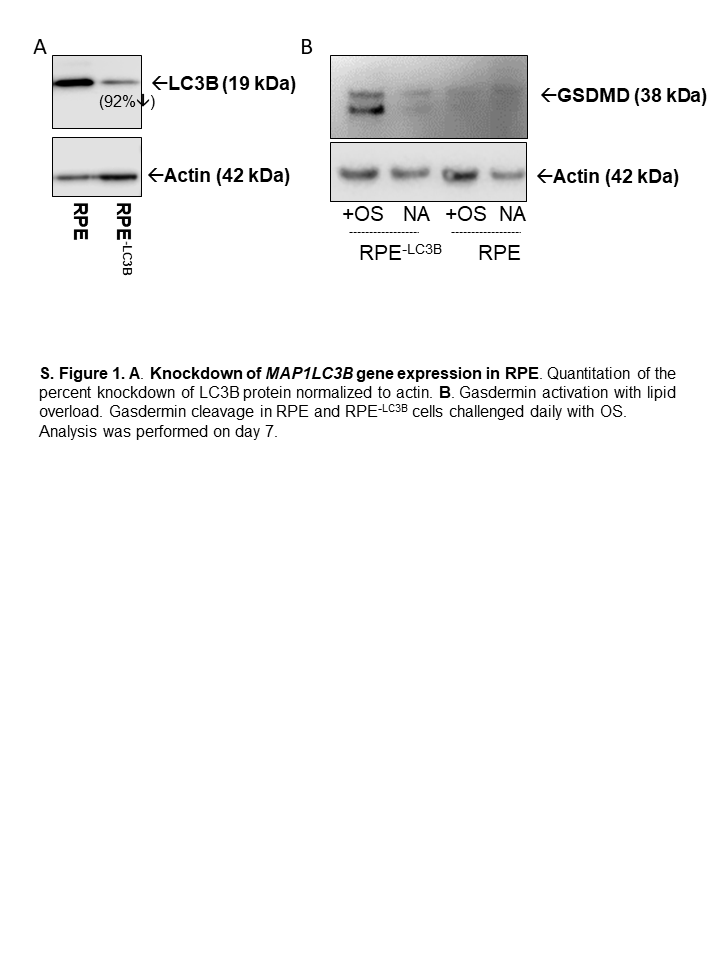

Supplement: Supplementary file 1 [file ijms-22-05764-s001.zip › S. Figure 1.TIF]

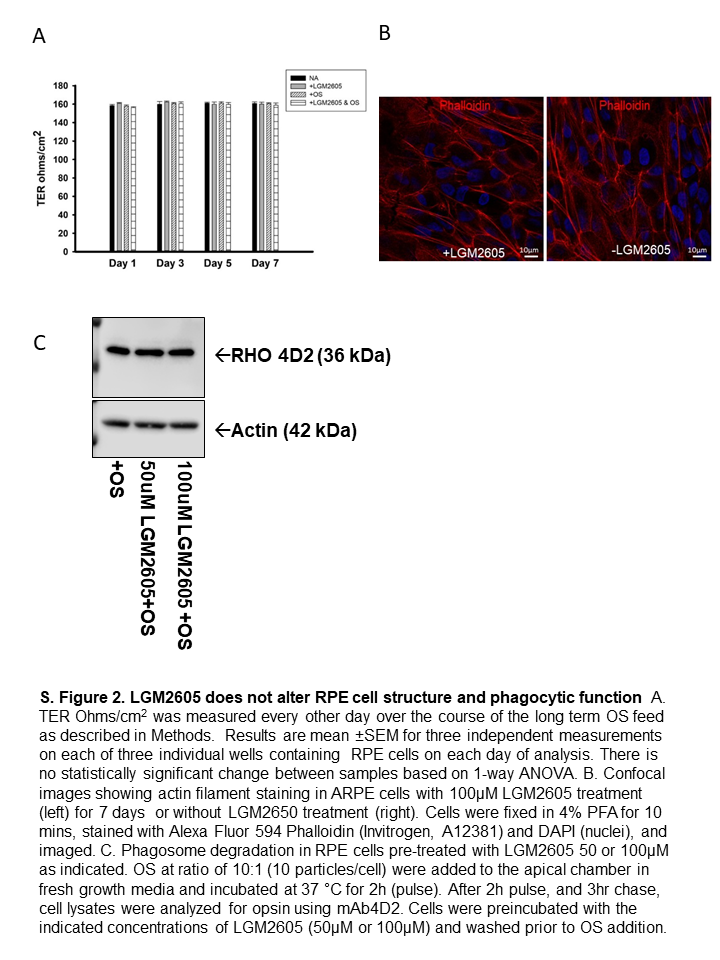

Supplement: Supplementary file 1 [file ijms-22-05764-s001.zip › S. Figure 2.TIF]

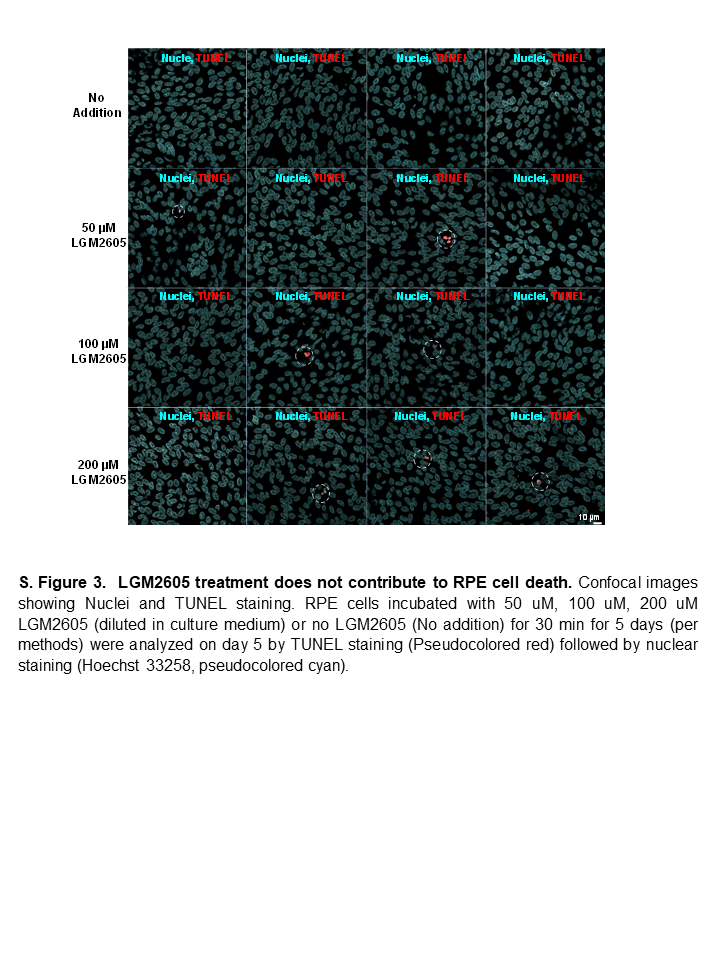

Supplement: Supplementary file 1 [file ijms-22-05764-s001.zip › S. Figure 3.TIF]

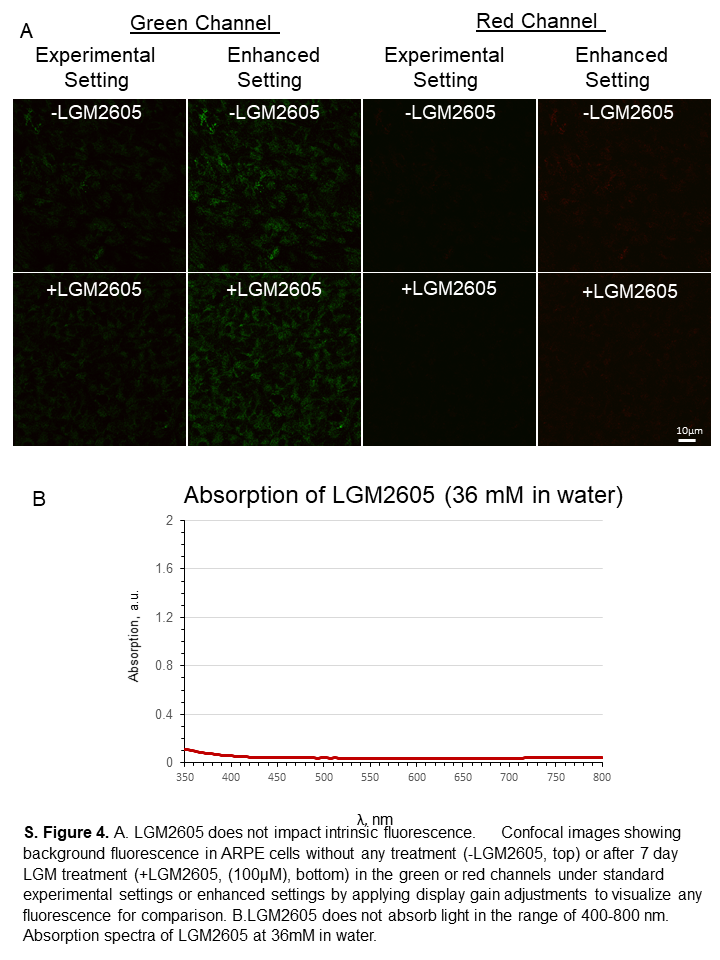

Supplement: Supplementary file 1 [file ijms-22-05764-s001.zip › S. Figure 4.TIF]
